# Spatial heterogeneity and socioeconomic determinants of opioid prescribing in England between 2015 and 2018

**DOI:** 10.1186/s12916-020-01575-0

**Published:** 2020-05-15

**Authors:** Rossano Schifanella, Dario Delle Vedove, Alberto Salomone, Paolo Bajardi, Daniela Paolotti

**Affiliations:** 1 Computer Science Department, University of Turin, Via Pessinetto 12, Turin, 10149 Italy; 2 ISI Foundation, Via Chisola, 5, Turin, 10126 Italy; 3 Department of Chemistry, University of Turin, Via Pietro Giuria, 7, Turin, 10125 Italy

**Keywords:** Opioid crisis, Prescribing data, Spatial analysis, Public health

## Abstract

**Background:**

Opioid overdoses have had a serious impact on the public health systems and socioeconomic welfare of several countries. Within this broader context, we focus our study on primary care opioid prescribing in England from 2015 to 2018, particularly the patterns of spatial variations at the community level and the socioeconomic and environmental factors that drive consumption.

**Methods:**

Leveraging open data sources, we combine prescription records with aggregated data on patient provenance and build highly granular maps of Oral Morphine Equivalent (OME) prescribing rates for Lower Layer Super Output Areas (LSOA). We quantify the strength of spatial associations by means of the Empirical Bayes Index (EBI) that accounts for geographical variations in population density. We explore the interplay between socioeconomic and environmental determinants and prescribing rates by implementing a multivariate logistic regression model across different temporal snapshots and spatial scales.

**Results:**

We observe, across time and geographical resolutions, a significant spatial association with the presence of localized hot and cold spots that group neighboring areas with homogeneous prescribing rates (e.g., EBI = 0.727 at LSOA level for 2018). Accounting for spatial dependency effects, we find that LSOA with both higher *employment deprivation* (OR = 62.6, CI 52.8–74.3) and a higher percentage of ethnically *white* (OR = 30.1, CI 25.4–35.7) inhabitants correspond to higher prescribing rates. Looking at educational attainment, we find LSOA with the prevalent degree of education being *apprenticeship* (OR = 2.33, CI 1.96–2.76) a risk factor and those with *level 4+* (OR = 0.41, CI 0.35–0.48) a protective factor. Focusing on environmental determinants, *housing* (OR = 0.18, CI 0.15–0.21) and *outdoor environment deprivation* (OR = 0.62, CI 0.53–0.72) indices capture the bi-modal behavior observed in the literature concerning rural/urban areas. The results are consistent across time and spatial aggregations.

**Conclusions:**

Failing to account for local variations in opioid prescribing rates smooths out spatial dependency effects that result in underestimating/overestimating the impact on public health policies at the community level. Our study suggests a novel approach to inform more targeted interventions toward the most vulnerable population strata.

## Background

Annual deaths related to drug overdoses have incessantly risen in the last decade in the USA, with more than 130 cases estimated to result from opioid overdoses every day [1]. This is seriously impacting the national public health system, as well as social and economic welfare, and seems to be the result of a triple wave epidemic of three classes of opioids: prescription pain relievers [2], heroin, and novel synthetic opioids such as fentanyl [3]. The issue dates back to the late 1990s when healthcare providers became increasingly willing to prescribe opioids for chronic pain with causes other than cancer. This led to a surge in the misuse of opioid medications before it became evident that they could be highly addictive. Many victims of opioid use disorder had their first experience with opioids through prescription drugs, which often led to addiction, and in cases of high dose opioid consumption, overdose and death [4, 5]. Access to medical opioids is often made even easier by the sharing of pharmaceuticals among friends and relatives [6].

Deaths related to opioid prescription misuse have also increased in several countries other than the USA. Indeed, in Western and Central Europe, the downward trend in opioid use observed since the beginning of the millennium came to an end in 2013 [7]. In particular, the United Kingdom (UK) has had a consistent upward trend in opioid prescribing [8, 9], and more than half of the individuals in treatment for any dependence in England have problems related to opioid consumption [10]. The number of drug misuse deaths related to opioid abuse has had a fourfold increase in England and Wales between 1993 and 2017, with a sharp rise between 2013 and 2015. In 2017 alone, there were about 2000 deaths [11]. Furthermore, it is likely that synthetic opioid-related deaths have been underestimated since many laboratories do not test for fentanyl or its analogs, and others lack the appropriate sensitivity or specificity for some of these highly potent emerging agents.

Some studies have shown that observed excesses in opioid prescribing are not a uniform phenomenon and they affect different regions of England with varying intensity [8, 12]. These studies have used official government information from the National Health Service (NHS), England’s primary care prescribing dataset, for opioid prescriptions in practices in England with the main goal of assessing long-term prescription trends and patterns of geographical variations. These analyses have focused on a practice perspective (i.e., practice-level data) with a large-scale geographical resolution of Clinical Commissioning Groups (CCG)—clinically led statutory bodies responsible for the planning of healthcare services for their local areas. England has more than 200 CCG altogether commissioning care for an average of 226,000 people each [13]. The main conclusions and recommendations of these works pertain to promoting best practice in chronic pain prescribing and reducing geographical variation, shifting the responsibility for opioid misuse entirely onto the practices. The thesis that the local prescribing variations may be attributable to clinician behavior is, indeed, shared by other working groups as well [2, 14, 15].

In this work, we adopt a complementary approach that focuses on the patients and the characteristics of the communities they live in, rather than targeting the prescribing procedures of the general practices alone. The goal is to shed light on potential sociodemographic factors associated with opioid consumption among the general population, zooming in from the macro-perspective of CCG-based studies to a finer geographical resolution. To this aim, we employ the NHS England primary care prescribing dataset to model spatial variations in opioid prescriptions at different spatial scales for the period 2015–2018. We adopt aggregated information in the administrative areas where the registered patients reside, and we develop a methodology to redistribute opioid consumption among these spatial units. We use governmental open data on sociodemographic indicators provided by the 2011 UK Census to explore potential determinants associated with the observed variations in space and time. We adopt a multivariate spatial regression model to account for spatial dependencies in the prescribing process.

The contribution of this approach is manifold; we (a) model the opioid prescribing patterns from the patient perspective rather than the general practice, (b) adopt a fine-grained spatial scale that could inform the design of public health policies at city or neighborhood levels, and (c) assess the impact of different spatial and temporal scales on the model stability.

## Methods

### Data

#### Drug prescriptions

Our primary source of information is the practice-level prescribing data [16] provided by the NHS and made available by the NHS Digital department^1^. The dataset covers NHS prescriptions written in England and dispensed in the community in the UK by general practitioners (GPs) and other non-medical prescribers (such as nurses and pharmacists) who are attached to practices. Prescriptions data have been published monthly since August 2010. Each record logs the total number of prescribed items where an item refers to a single supply of a medicine, dressing, or appliance, and the quantity expressed in units that depends on the formulation of the product, e.g., number of tablets, capsules, ampules, or milliliters of liquid, or grams of solid, like a cream. As suggested by the NHS, the raw item count might be a weak indicator of consumption as it does not provide information about the actual quantity of active ingredients prescribed.

The British National Formulary [17] (BNF) is a pharmaceutical reference book that contains a wide spectrum of information and advice on prescribing, along with specific facts about medicines available on the UK NHS. In this work, we refer to the BNF taxonomy to categorize medicines and appliances^2^ and to extract the data related to opioid prescriptions from the NHS open database. In particular, we refer to the paragraph Opioid Analgesics (4.7.2) in the BNF taxonomy.

#### Patient geographical provenance

NHS makes available aggregated data on the number of patients registered at each general practice stratified by gender and area of residence^3^. Data are provided quarterly and cover the period beginning in 2014. However, we excluded the 2014 records from our analysis due to incompatibilities between the boundaries of the administrative areas from the censuses in 2001 and 2011 that made the patient provenance not determinable for approximately 2 million individuals.

#### Spatial units

**Fig. 1:**
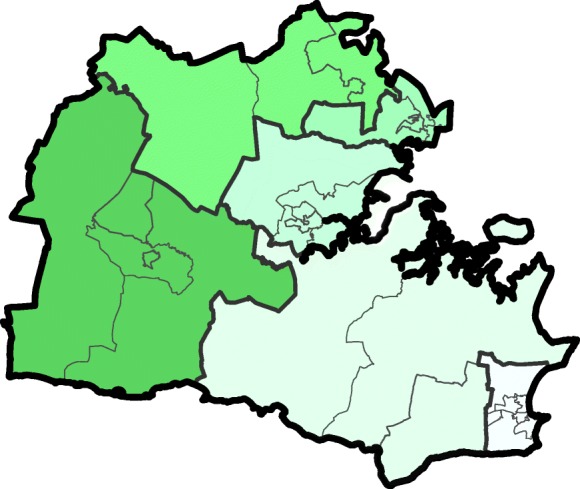
Hierarchical organization of the administrative spatial units in England. Schema of the multiple administrative resolutions of an exemplary LAD (Purbeck): the bold black line draws the external boundary, colored areas delimit the corresponding MSOA, and the internal borders in light gray define the fine-grained organization in LSOA

In this work, we adopt as reference the spatial units Lower Layer Super Output Areas (LSOA), which are administrative areas from the census corresponding to an average population of 1500 individuals. In 2018, England registered 32.844 LSOA. In contrast with previous work on prescribing patterns in the UK, LSOA provide a considerably finer granularity that enables community-level observations. Census regions in the UK are hierarchically organized in multiple resolution levels (see Fig. 1 for a visual representation). This allows us to replicate the study for coarser aggregations, namely the Middle Super Output Areas (MSOA), that have an average population of 7200 individuals [18], and the Local Authority Districts (LAD), that correspond to subnational divisions of England that are used for local government purposes [19]. The hierarchical structure and the shapefiles of the various census units are provided by the Open Geography Portal^4^ of the Office for National Statistics (ONS)^5^.

#### Socioeconomic indicators

The ONS is the largest independent producer of official statistics in the UK. It provides detailed and abundant socioeconomic, cultural, and demographic information about the population as measured by the census that was most recently updated in 2011 [20]. Among the wide variety of available data, we select a set of demographic and cultural variables such as age, gender, ethnicity, religion, and education attainment at the LSOA level. We normalize variables (in percentages) according to population or number of households in the reference spatial units.

In addition, we consider the Indices of Multiple Deprivation [21] (IMD), which are statistics on relative deprivation along the dimensions of income, employment, health, education, crime, housing, services accessibility, and environment. We note that the census and the IMD datasets have been collected in different years, 2011 and 2015, respectively, while prescription data are updated monthly. We thus consider the sociodemographic features as constant over the period of the study. Data are prepared and preprocessed with the *pandas* library in *Python* and organized in a *PostgreSQL* database.

### Mapping prescribing data into Oral Morphine Equivalent consumption rates

To represent opioid consumption at the population level, we refer to the Oral Morphine Equivalent [22] (OME) system that provides a correspondence between active substances in various opioid-based drugs and morphine. The use of the OME metric is preferable for opioid utilization studies as it facilities both interpretation and comparison between opioids and geographical locations accounting for the different concentration of active ingredients and drug presentations.

Each record in the prescribing data refers to a drug *d* identified by its BNF code and contains the quantity of units *q*_*d*_, e.g., the number of tablets, pills, or ampules, prescribed. From the drug name, we extract the quantity of active ingredient per milligram present in a unit of product. To derive the OME consumption *ome*(*g*) for a practice *g*, we adopt the methodology and the conversion table proposed in [23] and we sum the contribution of all units prescribed in *g*. A complete list of medicines together with the amount of active ingredients and their OME multipliers is provided in Additional file 1.

Extending previous work, we are interested in modeling opioid consumption from the end-user perspective and we hypothesize, therefore, that the prescriptions from a given practice are uniformly assigned to its registered patients. Since we know the geographical provenance of registered patients, we are able to redistribute the consumption flow among the spatial units. More formally, we define the OME consumption in an area *u* as:

1 $$ ome(u) = \sum_{g \in G_{u}} ome(u,g)  $$

where *G*_*u*_ is the set of practices with at least one patient living in *u* and *ome*(*u*,*g*) is the fraction of the overall consumption due to patients living in *u* and registered at *g*. Since we do not have prescription records at the level of individuals, *ome*(*u*,*g*) must be estimated. We use the following relation:

2 $$ ome(u,g) = ome(g) \frac{p(u,g)}{p(g)}  $$

where *p*(*g*) is the total number of patients registered at the practice *g* and *p*(*u*,*g*) represents the fraction that lives in the area *u*. Finally, we derive the OME prescribing rate *μ*_*ome*_(*u*) for a spatial unit *u* by normalizing the raw consumption *ome*(*u*) with the number of patients *p*(*u*) that live in *u* and are registered in any practice, as follows:

3 $$ \mu_{ome}(u) = \frac{ome(u)}{p(u)}  $$

**Fig. 2:**
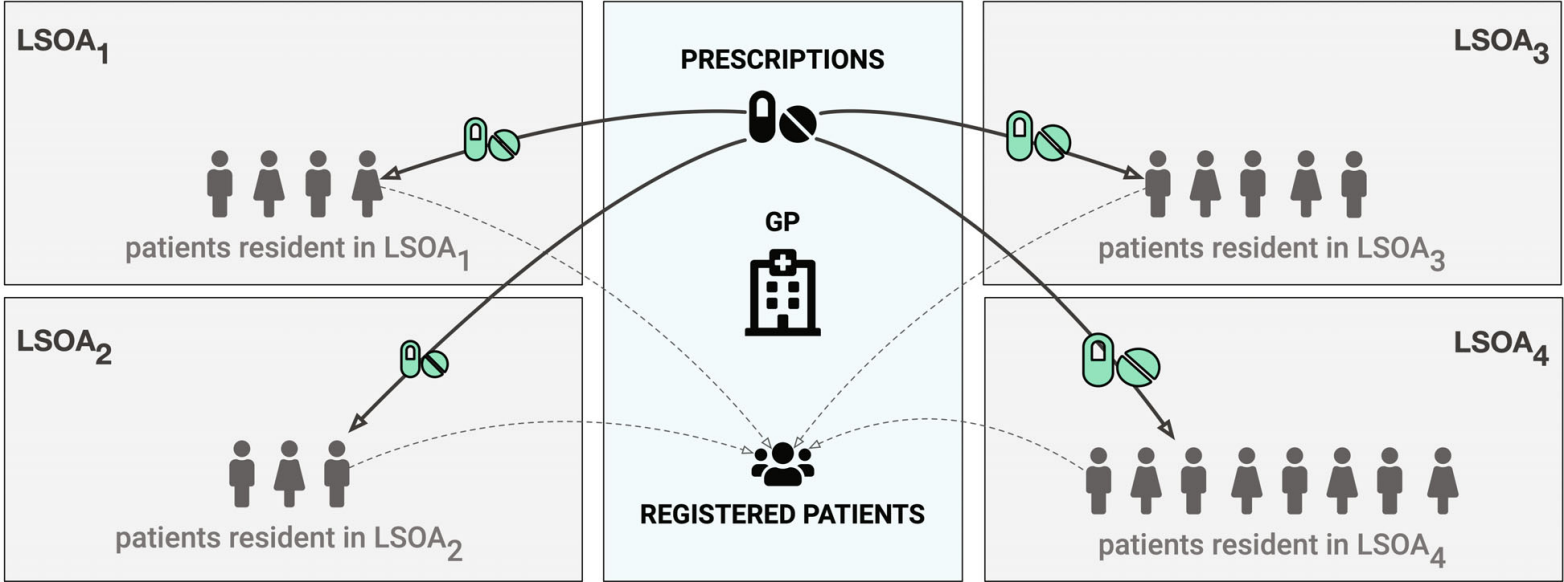
Prescribing geographical mapping. Schema of the drug redistribution method: prescriptions are spatially redistributed proportionally to the provenance of the patients registered at a practice

Figure 2 shows a visual representation of the prescribing rate construction methodology.

Since we estimate the number of patients living in a spatial unit *p*(*u*) by means of the patient provenance dataset, it is important to validate how accurately *p*(*u*) represents the actual population of the area *u* according to the official census. Therefore, we measure the Pearson correlation coefficients between the estimate and the official data across yearly time snapshots stratified by gender.

In this study, we focus on yearly temporal snapshots as aggregations of the original monthly prescribing data. Using a time trend analysis of the monthly opioid prescribing records for the period 2010–2018, we do not detect any relevant seasonal pattern to justify a finer temporal aggregation (see Additional file 2) [24, 25].

### Spatial analysis

To quantitatively assess global spatial dependency, we use the Empirical Bayes Index (EBI) [26]. EBI is an adjustment to Moran’s *I* autocorrelation coefficient [27] designed to overcome its variance instability due to varying populations across different spatial units. EBI is computed with a standardized version of rates, in order to make their variance uniform. We test the significance of EBI with a permutation test. Under the null assumption of spatial randomness, we generate an empirical distribution by permuting the rates among the spatial units. The number of permutation tests is set to 9999 and the significant p-level to 0.01. Local effects are estimated using the local version of the EBI as proposed in [26]. Statistics of local association rely on tests of spatial association for each location in the data, and the issues of multiple comparisons and dependent tests are a concern when assessing their significance [28, 29]. To address multiplicity, we implement the correction proposed in [30] based on the false discovery rate (FDR) and we control for the average rate that declarations of significance are truly non-significant. In the experimental phase, we run 9999 test iterations and set *α*=0.05 to estimate the *p* value cutoff. Empirical and simulative experiments [31] show the usefulness of this approach in comparison to more conservative methods like the Bonferroni and Sidak corrections [32]. We acknowledge that there is not a completely satisfactory solution to this problem or a rigorous general mathematical underpinning to the FDR-based corrections for applications in geography. However, local estimators can still be valuable in initial exploratory analyses to identify critical areas that might drive further investigations. In this work, we adopt this view and we use local measures in a descriptive rather than inferential framework. To model the neighboring relation between spatial units we refer to spatial weights [33]. We adopt a *contiguity* approach based on the binary *queen* criterion where *w*_*h*,*k*_=1 if the areas *h* and *k* share at least one vertex, 0 otherwise. To assess the sensitivity to variations of the spatial relations, we compute alternative weights measures, in particular, the *k-nearest neighbors (knn)* approach where each area has a fixed number of *k* closest neighbors. We adopt a Euclidean distance function computed between the centroids of the spatial units to rank areas by distance. Finally, we explore different weight strategies based on kernel functions with adaptive bandwidth, in particular, the uniform, and gaussian forms. We set the number of nearest neighbors *k*=[5,10,15,20] to estimate the bandwidth and the neighborhood size in the *knn* approach. For the analysis, we use the *pysal* library in *Python*.

### Analysis of determinants

To explore the potential socioeconomic and environmental factors associated with opioid analgesic prescribing patterns, we implement a predictive pipeline that involves feature normalization and selection steps, followed by a multivariate logistic regression analysis. First, we select sociodemographic indicators following the insights from previous studies [34–37], namely gender, age, ethnicity, educational attainment, population density, economic indicators, and deprivation-related variables. A description of the initial set of features can be found in Table 1. Second, we check for multicollinearity, and in the presence of strong correlations among a group of variables (*ρ*>0.9), we filter out the ones with less association with the outcome variable. Third, we apply a spatial lag transformation to the explanatory variables to incorporate the dependency from neighboring spatial units. Given a spatial unit *i* and a variable *y*, the spatially lagged version is computed as:

$$y_{lag_{i}} = \sum_{j} w_{ij} y_{i}.$$

This corresponds to the smoothing of the variable computed as a spatially weighted sum. We then discretize the candidate predictive lagged features in quintiles (i.e., we generate five categorical binary variables for each feature, according to observations within a specific quintile) and binarize the dependent variable using the median to discriminate between high and low prescribing rates. Fourth, we identify important variables with an influence on the outcome. We implement an *all-subsets* selection strategy using the Bayesian Information Criterion (BIC) [38]. Even though they are implemented extensively in practical scenarios, variable selection methods may suffer from model instability or potential bias in parameter estimates and confidence intervals. To estimate these effects, we study the stability to random perturbations of training samples using the methodology proposed in [39]. We implement a subsampling without replacement routine that randomly selects 63.2% of the initial datasets, and we run the selection procedure on the subsample. We select this threshold so that the number of observations is, on average, the same as the number of unique observations in a bootstrap pseudo-sample. The subsampling technique has been extensively studied, and it shows asymptotic consistency even in cases where the classical bootstrap fails [40]. We perform 200 subsampling iterations and compute the stability estimator proposed by Nogueira et al. [41] along with variable and model inclusion frequencies. After identifying the final set of covariates, we run a multivariate logistic regression using the area under the receiver operating characteristics curve (ROC AUC) to evaluate model performance in a cross-validation setting. We use a logit transformation to estimate the odds ratio along with 95% confidence interval (CI) for each of the categorized explanatory variables [42]. To test whether the estimated coefficients are statistically significant, we adopt the *Wald test* [43]. For the analysis, we use the *statsmod* and *scikit-learn* modules in *Python*.

**Table 1:** Data source and description for the explanatory variables

Variable	Type	Source	Description
Demography			
16–59 years	%	Census	Percentage of people between 16 and 59 years
60+ years	%	Census	Percentage of people with more than 60 years
Whites	%	Census	Percentage of people of white ethnicity
Asians	%	Census	Percentage of people of Asian ethnicity
Blacks	%	Census	Percentage of people of black ethnicity
Females	%	Census	Percentage of females
Economy			
Income	IMD	Government	Score of deprivation relating to low income
Employment	IMD	Government	Score of deprivation relating to exclusion from work (unemployment, sickness, disability)
Housing	IMD	Government	Score of deprivation relating to homelessness, house affordability, and overcrowding
Indoor env.	IMD	Government	Score of deprivation relating to Decent Homes Standard
Culture			
Christians	%	Census	Percentage of Christian people
Not Christians	%	Census	Percentage of people with religions other than Christianism
Atheists	%	Census	Percentage of people without religion
Young education	IMD	Government	Score of deprivation relating to education of children and young people
Adult education	IMD	Government	Score of deprivation relating to education of adults
No qualifications	%	Census	Percentage of people with no education degree ^∗^
Level 1	%	Census	Percentage of people with level 1 education degree (as highest certification) ^∗^
Level 2	%	Census	Percentage of people with level 2 education degree (as highest certification) ^∗^
Apprenticeship	%	Census	Percentage of people with an apprenticeship (as highest certification) ^∗^
Level 3	%	Census	Percentage of people with level 3 education degree (as highest certification) ^∗^
Level 4+	%	Census	Percentage of people with level 4 or higher education degree (as highest certification) ^∗^
Environment			
Crime	IMD	Government	Score of deprivation relating to violence, burglary, thefts, and criminal damages
Service accessibility	IMD	Government	Score of deprivation relating to physical proximity of local services
Outdoor env.	IMD	Government	Score of deprivation relating to air quality and traffic accidents

^*^[61]

## Results

### Mapping prescribing data into Oral Morphine Equivalent consumption rates

The proposed approach to estimate the prescribing rates at fine-grained administrative units provides an accurate picture of the spatial heterogeneity underpinning the phenomenon. Notably, since only 25% of LSOA in England contain general practices, characterizing prescribing patterns on this basis would limit our analysis to just this percentage. On the contrary, because patient geographical provenance covers England in full, with our method, we can assign an estimated rate to all LSOA (see Additional file 3 for more details). As expected, coverage improves when we aggregate to coarser spatial units such as CCG. Moreover, the spatial distribution of the patients registered to a practice shows a strong correlation with the population living in an area as gathered from the official census statistics. In fact, we observe Pearson’s correlation coefficient ranging from 0.8 and 1 depending on the spatial aggregation considered (see Table 2). The relation is consistent even if broken down by gender. Patient statistics slightly overestimate the number of inhabitants (less than 4%), both because an individual may be registered with more than one practice and because non-residents may be registered [44].

**Table 2:** Average correlation between inhabitants and registered patients in England

	LSOA	MSOA	LAD	CCG
Total	0.86	0.93	∼1	∼1
Males	0.81	0.92	∼1	∼1
Females	0.85	0.94	∼1	∼1

### Spatial analysis

**Fig. 3:**
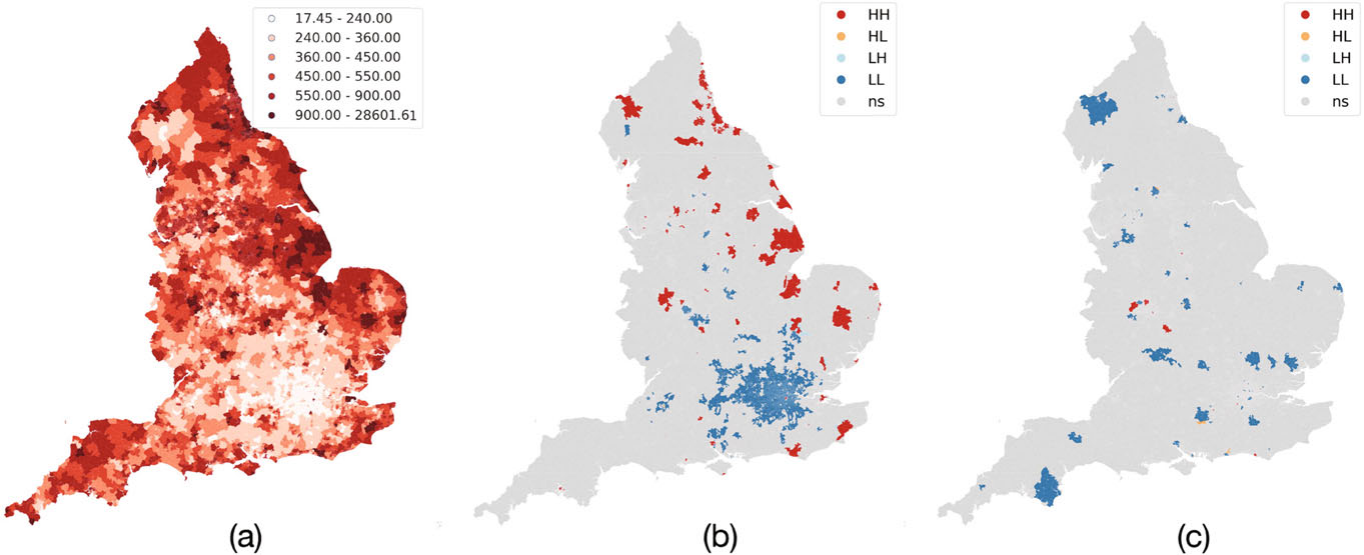
Spatial distributions at LSOA level for 2018: **a** OME rate, **b** hot/cold spots, and **c** hot/cold spots of the ratio between the OME rate in 2017 and 2018

To assess the variability of the target quantity *μ*_*ome*_, we compute its mean, variance, and quantiles across spatial units in the LSOA, MSOA, and LAD spatial aggregations and we observed that variability naturally decreases as the granularity gets coarser. Figure 3a shows the spatial distribution of the OME rates during 2018 for the LSOA. A darker color indicates higher prescription rates; the darkest red band represents the 95th percentiles of the distribution (spatial units with more than 900 mg OME per person). Qualitatively, the areas with the greatest consumption of opioid active substances are mainly concentrated along the eastern coast, e.g., in the counties of Kent and Sussex in the southeast, in extensive areas of the southwest, and in many zones across the Midlands and the northwest. The London area shows among the lowest prescribing rates. Quantitatively, we estimate the global spatial autocorrelation and we observe a EBI=0.727 (*p* value < 0.0001, *queen* weights) that confirms the presence of a statistically significant spatial clustering effect where high (low) consumption areas tend to be spatially contiguous to units with similar values. The significance and the strength of the spatial autocorrelation are fairly consistent across temporal snapshots (0.801, 0.694, and 0.723 for 2015, 2016, and 2017, respectively). We still observe significant spatial dependency effects zooming out to coarser geographical resolutions with diminishing strength (MSOA=0.472, LAD=0.252 for 2018, *p* value <0.0001).

Focusing on the local indicators of spatial associations, the LISA estimators are shown in Fig. 3b, where HH, LL, HL, and LH represent, respectively, the *high-high* (hot spots), *low-low* (cold-spots), *high-low* (areas with higher than average values surrounded by low value areas), and *low-high* (areas with lower than average values surrounded by high value areas). The cold spots in dark blue are observed in the London area and its closest counties, along with in delimited zones of Somerset, Wiltshire, Hampshire, Cumbria, Derbyshire, and West Midlands. On the contrary, hot spots in dark red that might indicate anomalies in population health or potential overprescription habits are mainly observed in the northeast and the eastern coast, with other limited areas across the northwest, West Midlands, East of England, Sussex, and Kent. Shifting the focus on the *high-low* and *low-high* cases, we observe the existence of, respectively, only 2 and 29 instances in 2018. However, further investigations are necessary to shed light on the presence of local variability, since it might hide unconventional prescription practices attributable to physicians or pharmacists. This is an issue for future research to explore.

To capture the spatio-temporal evolution, we compute the ratio between consumption rates at each spatial unit for pairs of consecutive years, which enables the investigation of local temporal patterns. Hot spots and cold spots of areas with the largest increase/decrease in opioid prescriptions between 2017 and 2018 are shown in Fig. 3c. While the majority of the spatial units does not register a significant variation, there are clusters of areas with evident decreasing rates, e.g., Cumbria, or increasing rates, e.g., small spots in West Midlands.

Additional file 4 contains a sensitivity analysis on how the choice of spatial weights affects the computation of the local measures of spatial association.

### Analysis of determinants

With the step of feature selection, we identify the following set of variables (in parenthesis, we report the sign of the correlation coefficient with the target variable *μ*_*ome*_): *employment* (+), *whites* (+), *housing* (−), *apprenticeship* (+), *outdoor environment* (−), and *level 4+* (−). We observe a high degree of stability according to the framework proposed in [41] (0.88 in 2018) and a variable selection frequency across subsampling iterations close to 1. The same pool of variables is selected also across the period 2015–2017, with the exception of *level 4+* in 2015 and 2016; however, educational attainment remains a relevant dimension by means of the presence of the *apprenticeship* variable.

Based on the selected lagged features, we train a logistic regression model for each year. We obtain an average AUC∼0.89 across years that denotes a solid performance. We report in Table 3 the interpretation of the logit regression model for 2018, along with the proportion of OME distributed in the LSOA belonging to the highest median and stratified by various explanatory variables. A thorough analysis and tentative interpretation of the relations identified are presented in the “Discussion” section.

**Table 3:** Median proportion of OME contained in LSOA grouped by quintiles (2018)

LSOA		
Quantile feature	High median (%)	Multivariable logit
Employment		
Least deprived	6.4	1 (ref)
.	11.6	2.46 (2.24–2.7)
.	14.1	5.48 (4.91–6.11)
.	17.1	14.91 (13.08–17.0)
Most deprived	22.1	62.62 (52.8–74.28)
Whites		
Lowest percentage	3.7	1 (ref)
.	9.4	4.29 (3.75–4.9)
.	14.6	8.59 (7.41–9.97)
.	19.2	15.63 (13.34–18.32)
Highest percentage	24.4	30.15 (25.43–5.73)
Level 4+		
Lowest percentage	24.2	1 (ref)
.	18.1	0.81 (0.73–0.9)
.	14.8	0.82 (0.73–0.92)
.	9.8	0.59 (0.52–0.67)
Highest percentage	4.4	0.41 (0.35–0.48)
Apprenticeship		
Lowest percentage	3.2	1 (ref)
.	10.8	1.49 (1.29–1.71)
.	16.6	1.73 (1.48–2.03)
.	19.4	2.09 (1.78–2.47)
Highest percentage	21.2	2.33 (1.96–2.76)
Housing		
Least deprived	17.4	1 (ref)
.	17.1	0.69 (0.64–0.76)
.	18.0	0.6 (0.54–0.66)
.	14.4	0.37 (0.33–0.41)
Most deprived	4.3	0.18 (0.15–0.21)
Outdoor env.		
Least deprived	16.7	1 (ref)
.	18.6	1.18 (1.07–1.29)
.	16.2	1.01 (0.91–1.12)
.	14.4	1.08 (0.97–1.21)
Most deprived	5.3	0.62 (0.53–0.72)

Odds ratio and 95% CI provided by the multivariable logistic regression

**Fig. 4:**
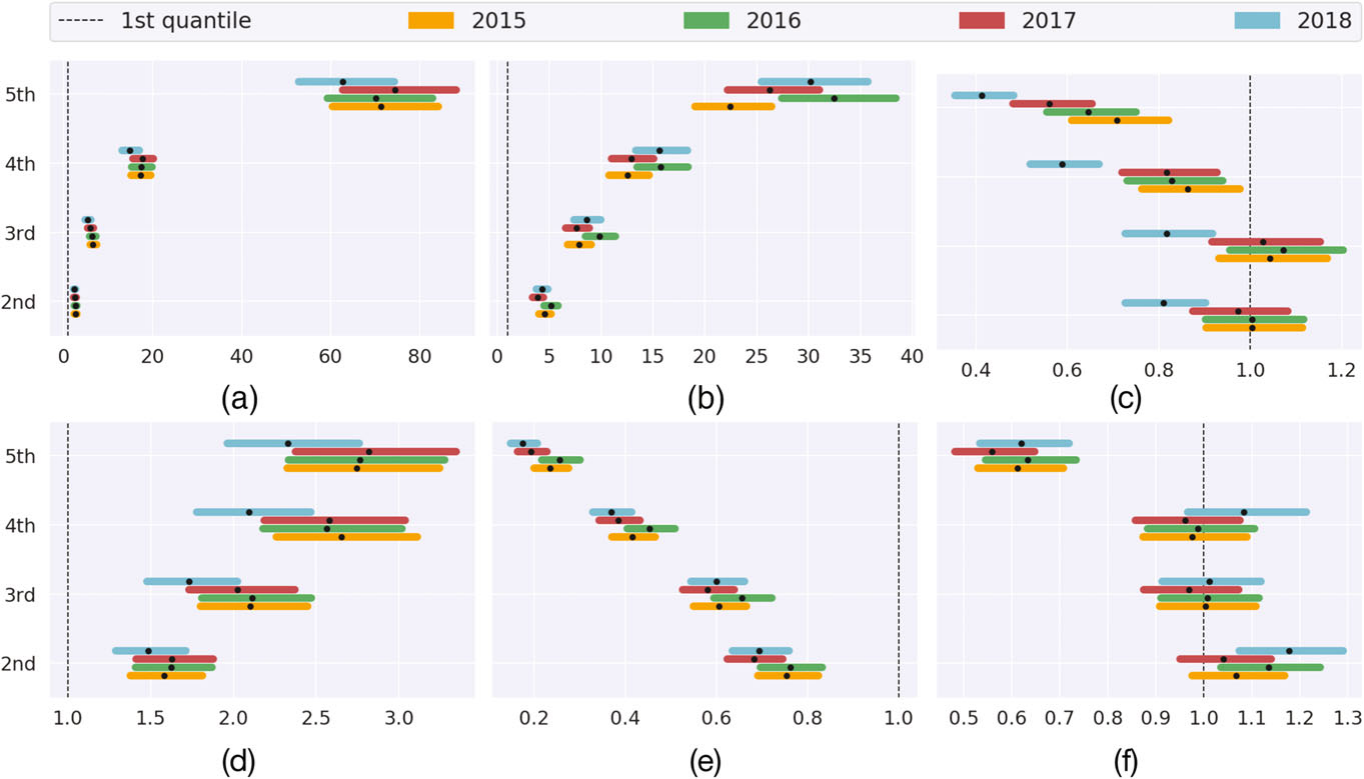
Odds ratio and 95% CI provided by the multivariable logistic regression at LSOA level for the predictors: **a** employment, **b** white, **c** level 4+, **d** apprenticeship, **e** housing, and **f** outdoor env.

Figure 4 summarizes the odds ratio of the multivariate logistic regression model grouped by year. We note that the odds ratios are fairly consistent across time (with a weaker agreement for the year 2018 that could potentially be due to the temporal misalignment with the census variables). Moreover, the overall trend seems significant for all the variables, with the exception of *outdoor environment* and *level 4+* for specific quintiles.

### Spatial scaling

**Fig. 5:**
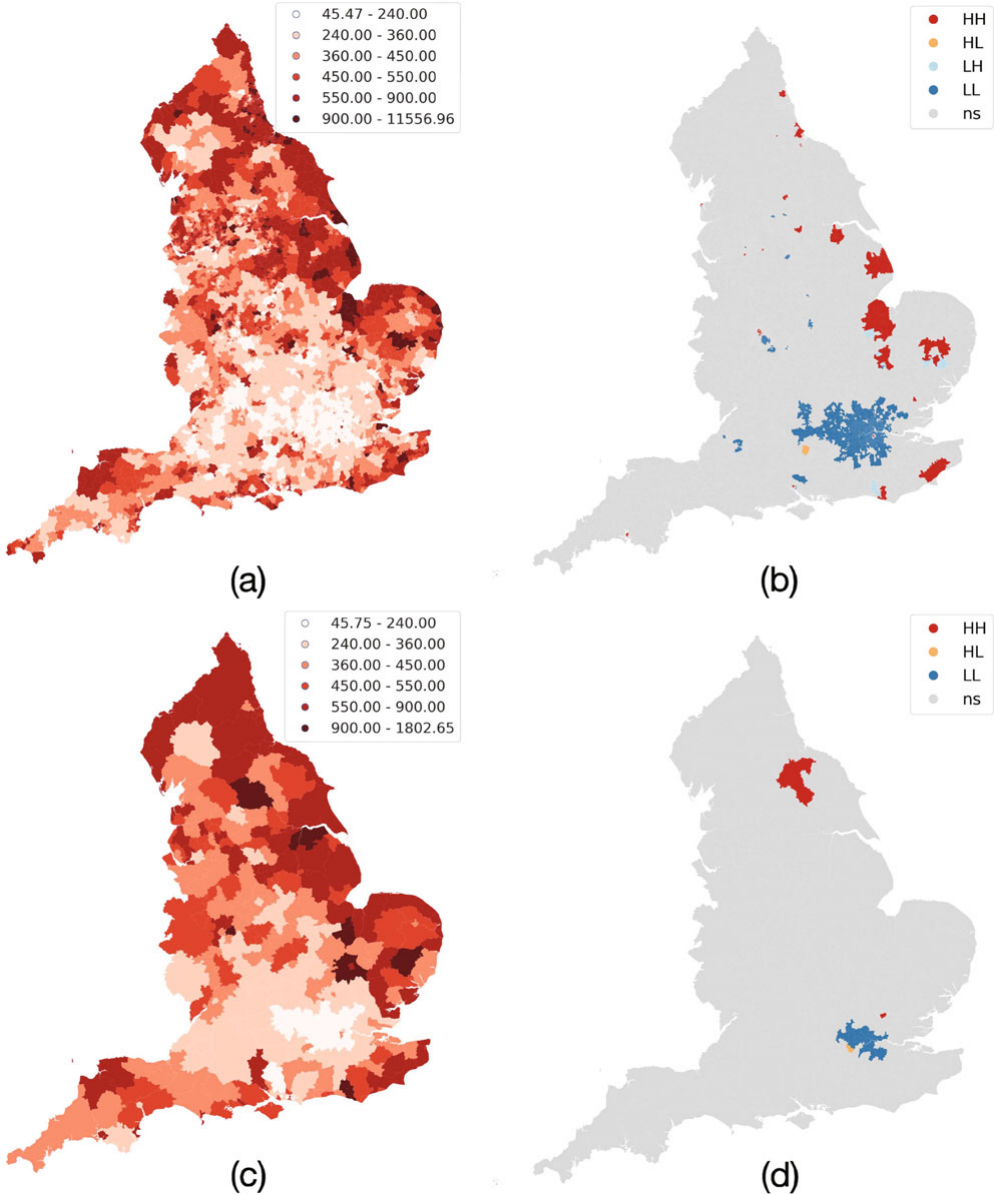
Spatial distributions at MSOA and LAD levels for 2018: **a** OME rate (MSOA), **b** hot/cold spots (MSOA), **c** OME rate (LAD), and **d** hot/cold spots (LAD)

To estimate the impact of scaling on spatial associations, we perform the same experimental pipeline for two coarser geographical resolutions: MSOA and LAD (see Additional file 5 for more details). When aggregated over larger spatial regions, normalized values tend to be smoothed toward the mean which hides relevant geographical heterogeneities as we report quantitatively in the “Discussion” section. The same holds for the identification of hot/cold spots, where the coarser granularity fails to identify some significant critical areas, as shown in Fig. 5b, d. Focusing on the MSOA use case, the feature selection routine identifies a coherent set of features, with the exception of *outdoor environment*. The selection is fairly consistent across years.

## Discussion

In this study, we focus on the spatial heterogeneity of opioid prescribing and, in particular, on the social determinants of health, i.e., the conditions in which people are born, grow, live, work, and age, which contribute to health inequities. Since substance abuse is strongly influenced by interpersonal, household, and group dynamics, the approach of analyzing dependencies at fine-grained spatial units provides the tools to monitor social and environmental risk factors of local communities in juxtaposition to a regional or national scope. Consistent with the literature on opioid abuse, we draw a connection with a multidimensional range of determinants (see Table 3).

Economic hardship and high rates of unemployment have been extensively associated with communities hit hard by the opioid crisis such as Appalachia and urban centers in the USA [45], as well as Russian communities dislocated by the Soviet Union’s economic collapse [46]. Employment deprivation is also confirmed to have the strongest effect in the England scenario, with the most deprived areas having more than 60 times higher probability to experience a high consumption rate (consumption in the high median of the distribution) than the least deprived.

A literature review to evaluate minority racial disparities in opioid-containing compound use, abuse, and care in the USA shows the population of white individuals being prescribed at double the rate [47, 48] of non-white minorities. We observe a similar trend in England, where the variable accounting for the percentage of residents of the white British ethnicity scores as the second most predictive determinant. Historical and cognitive biases along with limited access to treatment may insulate non-white minorities. Moreover, physician bias [36, 49, 50], media portrayal of abuse disorders, and governmental regulation are widely mentioned as a polyfactorial root of racial inequity in the opioid epidemic [47]. Ethnicity has been connected in the past with the nature of opioid compounds consumed; where prescriptions have been mostly connected to the white community, heroin, synthetic, and home-produced substances have been mostly connected to ethnic minorities. Understanding ethnicity differences in long-term trends by opioid type may contribute to improved public health interventions.

A wide body of literature connects educational attainment to opioid consumption. In [51], the authors evaluate opioid prescription rates in the context of pain treatment in emergency departments. They observe that after adjusting for age, gender, income, and pain severity, patients with the highest educational attainment are three times less likely to receive opioids than low-education patients. This relationship has been consistently observed outside the acute pain setting [52], along with the influence on healthy habits [53] and long-term health [54]. Factors such as patient understanding of their condition and self-efficacy of recovery, reduced pain catastrophizing, lower distress levels, and greater fear of addiction have been indicated as traits of highly educated patients [51]. On the other side, it has been hypothesized that physician prescribing behavior adapts according to patient socioeconomic status. This implies a shift in focus from patients being aware and willing to accept a treatment medication to physicians being willing to prescribe one. These results are consistent with our observations: higher educational attainment (*level 4+*) corresponds to a lower incidence of OME consumption. Similarly, the percentage of population with an apprenticeship level education (*apprenticeship*) is positively connected to OME consumption, probably due to the on-the-job nature of the training program, a higher incidence of serious accidents, or the more physically strenuous work conditions.

Health outcomes have also been associated with the environmental characteristics of the neighborhood where patients live. In [55], the authors show the interplay between neighborhood disadvantage and drug abuse, controlling for individual-level socioeconomic status. Looking at the problem from a different angle, this relationship has been indirectly explained by the increased social stresses and higher levels of psychological distress present among residents of disadvantaged neighborhoods [56]. Decades of research using animal models confirm the importance of stress in increasing the risk of substance abuse: in an environment without opportunities for play, exploration, and exercise, rodents have been shown to be more sensitive to the rewarding effects of heroin as compared to those in more enriched environments [57]. In our study, we find that higher values for the deprivation index *housing*, a composite measure related to house affordability, household overcrowding, and homelessness, correspond to a decrease in OME consumption rates. We speculate that the explanation for the counterintuitive association is multifold. First, the *housing* indicator blends into a single measure a wide range of determinants associated with the urban housing ecosystem. More importantly, the housing deprivation index is significantly correlated with the spatial distribution of urban-rural areas. We test this by assigning to each spatial unit a score from large rurality to full urbanity, then computing Spearman’s rank correlation coefficient with the housing variable categorized in quintiles. The results show a positive correlation (*ρ*=0.41, *p*<0.001) that indicates how *housing* captures the classification of LSOA in rural-urban areas. In other words, spatial units with higher housing deprivation belong on average to an urban context and vice versa. This observation leads us to connect rural-urban areas with respectively higher-lower OME consumption rates, which is consistent with several previous studies [8, 37, 58]. A similar case can be made for the *outdoorenvironment* indicator that combines a measure of air quality based on emission rates for four pollutants and road traffic accidents involving injury to pedestrians and cyclists. As expected, this deprivation index tends to have higher values in an urban setting (*ρ*=0.58, *p*<0.001). We note that the behavior of *outdoorenvironment* is the less stable among the determinants and has a significant role only for the highest quantile.

As shown in Fig. 4, the results discussed in the first part of this section are consistent across the temporal span of our analysis (2015–2018), which underlines that social and environmental determinants characterize the opioid prescribing patterns in England. We observe a small deviation in the intensity of some determinants in 2018 that is likely connected to the temporal mismatch with the socioeconomic dataset (2011 and 2015). Another important observation is related to the stability of the results when zooming out to the coarser spatial granularity of MSOA, which shows a good degree of robustness to the scaling problem that has been extensively studied in spatial analysis and behavioral geography [59].

### Limitations

The proposed methodology has some limitations. First, prescribing data are collected on a monthly basis that allows us to monitor the long-term trends in drug consumption. However, the socioeconomic indicators in Table 1 come either from the UK Census that is performed every 10 years (the last instance was in 2011) or from the Indices of Multiple Deprivation of 2015, which are computed every 4 or 5 years. This produces a mismatch between the temporal evolution of the target variable and the explanatory variables that are fixed across the period of study. However, the consistency of the results across the years 2015–2018 (see Fig. 4) provides a hint that despite the rapid changes of modern society, we are able to capture the underlying phenomenology with a sufficient degree of approximation. Second, the opioid consumption rates are computed under the hypothesis that the prescriptions filled by a practice are redistributed proportionally to its patients’ provenance areas. We argue that this hypothesis is reasonable due to the way in which LSOA are defined as groups of contiguous output areas with similar population size. Moreover, the fact that we are able to identify determinants that have been extensively discussed in previous work is indirect evidence of the reliability of our results. In future work, we plan to quantitatively validate our approach with real data from hospitals or diagnostic medical centers. Due to the high risk of (fatal) overdose, we will also try to compare spatial distributions for prescriptions to the geographical distribution of opioid-related deaths. Third, prescribing records are only a channel to shed light on the determinants of opioid abuse. In the case of the USA [57], for example, several distinct, well-established markets for opioids exist with overlapping demand. The products they supply include opioids prescribed, dispensed, and used by patients as medically intended; those prepared as a prescription but not used as intended, including opioids dispensed and misused, as well as those that are diverted before being dispensed (i.e., diverted from lawful channels of commercial distribution, such as wholesalers and pharmacies); and those supplied by drug trafficking organizations, mostly from international sources. Conditions appear ripe for fentanyl and counterfeit prescription pills to continue to spread, with potential effects not only on heroin and other illicit drug markets but also on markets for diverted prescription drugs. In this direction, we plan to complement the current study with analysis of alternative data sources, e.g., social media platforms like Reddit, that have recently been used to characterize the spatial distribution of the discourse around opioids in the USA [60]. Fourth, we merge data from all opioid prescriptions into one category by means of the OME transformation. While this approach proves effective to monitor a general trend in opioid abuse, a future development of our research will aim to pull out information related to every single molecule. Finally, our work falls in the category of an observational study and we do not explore any causal aspects. The socioeconomic and environmental determinants might actually be the result, instead of the direct cause, of the observed opioid consumption patterns.

## Conclusions

While previous work focuses on the general practice factors associated with opioid prescribing using coarse spatial aggregations, e.g., CCG [8, 12], one of the main contributions of this work is to shift the attention to patient provenance by proposing a methodology to redistribute the prescriptions generated by practices to LSOA where the patients reside. This enables us to shed light on the spatio-temporal patterns of opioid prescribing at an unprecedented spatial scale, and it has the potential to inform public health agencies of local effects and to support the design of more targeted and effective interventions. We note that previous studies based on CCG [8, 12] have been able to detect consumption patterns that are comparable to the output of our methodology to a large extent. In fact, Spearman’s rank correlation coefficient between the OME consumption rates for CCG as computed with the traditional practice-centric approach and the aggregation of CCG rates as computed after our patient-centric redistribution methodology is close to 1 (*ρ*=0.96, *p*<0.001 for 2015–2017 and *ρ*=0.95, *p*<0.001 for 2018). This validates our approach and shows how it is able to reproduce the geographical patterns observed in the literature.

To further explore the advantages and opportunities of working on a local scale, we offer an example. Even though between 1998 and 2016, opioid prescriptions increased by 34% in England (127% when accounting for the total oral morphine equivalency [8]), with the introduction of the Opioids Aware Resource^6^ in 2016, the volume of prescriptions dropped slightly, showing a reduction during 2016–2018. Aggregated statistics hide the heterogeneity of the spatial patterns where localized hot spot areas emerge (see red areas in Fig. 3c). For instance, consider the Local Authority District of Sandwell, a metropolitan borough in the West Midlands. In the years 2016–2018, it experienced an overall increase of 70 mg/year OME per patient. However, looking at a finer spatial granularity, the picture is quite a bit more complex: only 40 out of 186 LSOA had an increase greater than or equal to 70 mg/year OME, with 11 LSOA that experienced an abrupt growth above 600 mg/year OME and a peak around 2500 mg/year OME. We note that the areas with the highest increase are geographically clustered in the northern part of Sandwell, underscoring the importance of spatial analysis tools to properly model spatial dependency. On the other hand, the majority of Sandwell LSOA (100 out of 186) experienced a reduction in OME prescribing rates, while a handful of areas observed no significant changes.

We argue that policymakers could benefit from a methodology that allows fine-grained spatial monitoring to target effectively criticalities in local communities that would fade away with aggregation. We envision an integrated approach in which the two perspectives are intertwined in a data-driven framework able to capture these complementary facets and to support the design of effective preventive strategies.

## Supplementary information

**Additional file 1** List of medicines, amount of active ingredients and their OME multipliers.

**Additional file 2** Temporal analysis of the yearly OME prescribing rate.

**Additional file 3** Spatial distribution of the general practices in England at LSOA level.

**Additional file 4** Sensitivity analysis of the local measures of spatial association according to different weighting schemes.

**Additional file 5** Analysis of determinants at MSOA level (2018).

## Data Availability

The datasets used and analyzed during the current study are available from the corresponding author on reasonable request.

## Footnotes

1 https://digital.nhs.uk/

2 https://ebmdatalab.net/prescribing-data-bnf-codes/

3 https://digital.nhs.uk/data-and-information/publications/statistical/patients-registered-at-a-gp-practice

4 http://geoportal.statistics.gov.uk

5 https://www.ons.gov.uk/

6 https://fpm.ac.uk/faculty-of-pain-medicine/opioids-aware

## References

[1] CDC/NCHS, National Vital Statistics System, Mortality. CDC WONDER, Atlanta: GA: US Department of Health and Human Services, CDC. https://wonder.cdc.gov. Accessed 15 Apr 2020.

[2] Dhalla IA, Persaud N, Juurlink DN. Facing up to the prescription opioid crisis. BMJ. 2011;343. https://doi.org/10.1136/bmj.d5142. 10.1136/bmj.d5142 21862533

[3] Ciccarone D. The triple wave epidemic: supply and demand drivers of the US opioid overdose crisis. Int J Drug Policy. 2019. 10.1016/j.drugpo.2019.01.010. 10.1016/j.drugpo.2019.01.010 PMC6675668 30718120

[4] Cicero TJ, Ellis MS, Surratt HL, Kurtz SP (2014). The changing face of heroin use in the United States: a retrospective analysis of the past 50 years. JAMA Psychiatry.

[5] Bohnert AS, Valenstein M, Bair MJ, Ganoczy D, McCarthy JF, Ilgen MA, Blow FC (2011). Association between opioid prescribing patterns and opioid overdose-related deaths. JAMA.

[6] Lankenau SE, Teti M, Silva K, Jackson Bloom J, Harocopos A, M. T (2012). Initiation into prescription opioid misuse amongst young injection drug users. Int J Drug Policy.

[7] UNODOC. World drug report 2018; 2018, pp. 53–76. https://www.unodc.org/wdr2018/. Accessed 15 Apr 2020.

[8] Curtis HJ, Croker R, Walker AJ, Richards GC, Quinlan J, B. G (2018). Opioid prescribing trends and geographical variation in England, 1998–2018: a retrospective database study. Lancet Psychiatr.

[9] Zin CS, Chen LC, D. KR (2014). Changes in trends and pattern of strong opioid prescribing in primary care. Eur J Pain.

[10] Knight J, Brand P, Willey P, van der Merwe J. Adult substance misuse statistics from the national drug treatment monitoring system - 1 april 2017 to 31 march 2018: PHE Publications; 2017, pp. 44–50. https://www.gov.uk/government/statistics/substance-misuse-treatment-for-adults-statistics-2017-to-2018. Accessed 15 Apr 2020.

[11] ONS - deaths related to drug poisoning in England and Wales: 2017 registrations. https://www.ons.gov.uk/peoplepopulationandcommunity/birthsdeathsandmarriages/deaths/bulletins/deathsrelatedtodrugpoisoninginenglandandwales/2017registrations. Accessed 15 Apr 2020.

[12] Mordecai L, Reynolds C, Donaldson LJ, de C Williams A C (2018). Patterns of regional variation of opioid prescribing in primary care in england: a retrospective observational study. Br J Gen Pract.

[13] NHS - About CCGs. https://www.nhscc.org/ccgs/. Accessed 15 Apr 2020.

[14] Foy R, Leaman B, McCrorie C, Petty D, House A, Bennett M, Carder P, Faulkner S, Glidewell L, West R. Prescribed opioids in primary care: cross-sectional and longitudinal analyses of influence of patient and practice characteristics. BMJ Open. 2016;6(5). 10.1136/bmjopen-2015-010276. 10.1136/bmjopen-2015-010276 PMC4874107 27178970

[15] Meisenberg BR, Grover J, Campbell C, Korpon D. Assessment of opioid prescribing practices before and after implementation of a health system intervention to reduce opioid overprescribing. JAMA Netw Open. 2018;1(5). 10.1001/jamanetworkopen.2018.2908. 10.1001/jamanetworkopen.2018.2908 PMC6324493 30646184

[16] NHS - Practice Level Prescribing Data. https://digital.nhs.uk/data-and-information/areas-of-interest/prescribing/practice-level-prescribing-in-england-a-summary. Accessed 15 Apr 2020.

[17] BNF Booklet 76. https://www.bnf.org. March-September 2018.

[18] ONS. National statistics postcode lookup user guide (february 2018). 2018. http://geoportal.statistics.gov.uk/datasets/4ca06fae243147efb3df8a704653a99f Accessed 15 Apr 2020.

[19] Park N. Population estimates for the uk, england and wales, scotland and northern ireland: mid-2017. 2018:14. https://www.ons.gov.uk/peoplepopulationandcommunity/populationandmigration/populationestimates/bulletins/annualmidyearpopulationestimates/mid2017. Accessed 15 Apr 2020.

[20] Census - 2011 Census Data On Nomis. https://www.nomisweb.co.uk/census/2011. Accessed 15 Apr 2020.

[21] Smith T, Noble M, Noble S, Wright G, McLennan D, Plunkett E. The English indices of deprivation 2015 - technical report. 2015. https://www.gov.uk/government/publications/english-indices-of-deprivation-2015-technical-report. Accessed 15 Apr 2020.

[22] Nielsen S, Degenhardt L, Hoban B, Gisev N (2016). A synthesis of oral morphine equivalents (ome) for opioid utilisation studies. Pharmacoepidemiol Drug Saf.

[23] Curtis HJ, Croker R, Walker AJ, Richards GC, Quinlan J, B G. Supplementary appendix - opioid prescribing trends and geographical variation in England, 1998–2018: a retrospective database study. 2018. 10.1016/S2215-0366(18)30471-1. 10.1016/S2215-0366(18)30471-1 30580987

[24] Athanasopoulos G, Hyndman RJ. Forecasting: principle and practice: O Texts; 2018. http://OTexts.com/fpp2/. Accessed 15 Apr 2020.

[25] Dickey DA, Fuller WA (1979). Distribution of the estimators for autoregressive time series with a unit roots. J Am Stat Assoc.

[26] Assunção R, EA R (1999). A new proposal to adjust Moran’s I for population density. Stat Med.

[27] Moran PAP. Notes on continuous stochastic phenomena. Inst Stat Oxford Univ. 1950. 10.2307/2332142. 15420245

[28] Miller RG (1981). Simultaneous Statistical Inference, Springer series in statistics.

[29] Tukey JW (1991). The philosophy of multiple comparisons. Stat Sci.

[30] Benjamini Y, Hochberg Y (1995). Controlling the false discovery rate: a practical and powerful approach to multiple testing. J R Stat Soc Ser B (Methodol).

[31] Caldas de Castro M, Singer BH (2006). Controlling the false discovery rate: a new application to account for multiple and dependent tests in local statistics of spatial association. Geogr Anal.

[32] Anselin L (1995). Local indicators of spatial association-lisa. Geogr Anal.

[33] Rey SJ, Anselin L (2014). Modern spatial econometrics in practice: a guide to GeoDa, GeoDaSpace and PySAL.

[34] Dasgupta N, Beletsky L, Ciccarone D (2018). Opioid crisis: No easy fix to its social and economic determinants. Am J Public Health.

[35] Birnbaum HG, White AG, Schiller T, Waldman M, Cleveland JM, Roland CL (2015). Societal costs of prescription opioid abuse, dependence, and misuse in the United States. Pain Med.

[36] Netherland J, Hansen H (2017). White opioids: pharmaceutical race and the war on drugs that wasn’t. Biosocieties.

[37] Keyes KM, Cerdá M, Brady JRJE, Havens SG (2014). Understanding the rural–urban differences in nonmedical prescription opioid use and abuse in the United States. Nurs Stand.

[38] Schwarz G (1978). Estimating the dimension of a model. Ann Stat.

[39] Sauerbrei W, Buchholz A, Boulesteix A-L, Binder H (2015). On stability issues in deriving multivariable regression models. Biom J.

[40] Chernick MR (2011). Bootstrap methods: a guide for practitioners and researchers, Wiley Series in Probability and Statistics.

[41] Nogueira S, Sechidis K, Brown G (2018). On the stability of feature selection algorithms. J Mach Learn Res.

[42] Peng CJ, Lee KL, Ingersoll G (2002). An introduction to logistic regression analysis and reporting. J Educ Res.

[43] Hauck WW, Donner A (1977). Wald’s test as applied to hypotheses in logit analysis. J Am Stat Assoc.

[44] NHS - How to Register with a GP Practice. https://www.nhs.uk/using-the-nhs/nhs-services/gps/how-to-register-with-a-gp-practice/. Accessed 15 Apr 2020.

[45] Case A, Deaton A (2015). Rising morbidity and mortality in midlife among white non-Hispanic Americans in the 21st century. Proc Natl Acad Sci.

[46] Brainerd E, Cutler DM (2005). Autopsy on an empire: understanding mortality in Russia and the former Soviet Union. J Econ Perspect.

[47] Santoro TN, Santoro JD (2018). Racial bias in the us opioid epidemic: a review of the history of systemic bias and implications for care. Cureus.

[48] Alexander MJ, Kiang MV, Barbieri M (2018). Trends in black and white opioid mortality in the United States, 1979-2015. Epidemiol (Cambridge, Mass.).

[49] Green CR, Ndao-Brumblay SK, West B, Washington T (2005). Differences in prescription opioid analgesic availability: comparing minority and white pharmacies across Michigan. J Pain.

[50] Mazer-Amirshahi M, Mullins PM, Rasooly I, van den Anker J, Pines JM (2014). Rising opioid prescribing in adult U.S. emergency department visits: 2001–2010. Acad Emerg Med.

[51] More educated emergency department patients are less likely to receive opioids for acute pain. Pain. 2012; 153(5):967–73. 10.1016/j.pain.2012.01.013 PMC3334443 22386895

[52] Krebs EE, Lurie JD, Fanciullo G, Tosteson TD, Blood EA, Carey TS, Weinstein JN (2010). Predictors of long-term opioid use among patients with painful lumbar spine conditions. J Pain Off J Am Pain Soc.

[53] Winkleby MA, Jatulis DE, Frank E, Fortmann SP (1992). Socioeconomic status and health: how education, income, and occupation contribute to risk factors for cardiovascular disease. Am J Public Health.

[54] Pinsky JL, Leaverton PE, Stokes J (1987). Predictors of good function: the Framingham study. J Chronic Dis.

[55] Boardman JD, Finch BK, Ellison CG, Williams DR, Jackson JS (2001). Neighborhood disadvantage, stress, and drug use among adults,. J Health Soc Behav.

[56] Boardman JD (2004). Stress and physical health: the role of neighborhoods as mediating and moderating mechanisms,. Soc Sci Med.

[57] Eitan S, Emery MA, Bates MLS, Horrax C (2017). Opioid addiction: who are your real friends?. Neurosci Biobehav Rev.

[58] Cicero TJ, Surratt H, Inciardi JA, A. M (2007). Relationship between therapeutic use and abuse of opioid analgesics in rural, suburban, and urban locations in the United States. Nurs Stand.

[59] Watson MK (1978). The scale problem in human geography. Geografiska Ann Ser B Hum Geogr.

[60] Balsamo D, Bajardi P, Panisson A (2019). Firsthand opiates abuse on social media: monitoring geospatial patterns of interest through a digital cohort. The World Wide Web Conference, WWW ’19.

[61] ONS. 2011 census - glossary of terms. 2014. https://www.ons.gov.uk/census/2011census/2011censusdata/2011censususerguide/glossary. Accessed 15 Apr 2020.

