# Supplementary material for: Spatial heterogeneity and socioeconomic determinants of opioid prescribing in England between 2015 and 2018
Source: BMC Med. 2020 May 15;18:127. doi: 10.1186/s12916-020-01575-0 (PMC7227089; doi:10.1186/s12916-020-01575-0)
Supplement: Supplementary file 2 — Additional file 2 Temporal analysis of the yearly OME prescribing rate. [file 12916_2020_1575_MOESM2_ESM.pdf]

Additional File 2

A time trend analysis of monthly opioid prescribing rates for the period 2010-2018

Methods

We focus on the analysis of the temporal evolution of the monthly prescribing rates to (a) compare our analytical framework with previous work on opioid usage modeling in England, (b) to observe the presence of seasonality effects, and (c) to evaluate the appropriateness of the temporal granularity adopted in the spatial analysis (e.g., month, quarter, semester, or year). To this extent, we decomposed the time series assuming an additive model of the time series, that is

$$Y_t = T_t + S_t + e_t$$

where $\{T_t\}_t$ is the trend, $\{S_t\}_t$ the seasonality, and $\{e_t\}_t$ is the series of residuals. The trend component was first computed by applying a convolution filter to the data, calculated by a moving average formula. After having removed the trend values from the time series, seasonality was computed by averaging the detrended values. Removing also the obtained seasonality, residuals time series returned [24]. We checked the stationarity of $\{e_t\}_t$ to validate the meaningfulness of the decomposition by the Dickey-Fuller Test [25].

For analyses, we used *tsa* (time series analysis) module of *statsmodel* in *Python*.

Results

In order to understand what temporal granularity was suitable for studying the process of prescribing, we decomposed the time series of the volume of prescriptions and the OME amount in trend, seasonality and residuals. In both cases, no clear seasonality was revealed. Moreover, the fact that both the time series of residuals were stationary with a confidence of 99% implied the fact that the decompositions were reliable [24]. Consequently, we decided to opt in for yearly temporal snapshots. Consistently with previous studies, a nonnegligible increase in opioids prescription items was observed. During the time frame of interest, the trend in terms of pure prescribing volume increased of 26.6% (from $1.53 \cdot 10^6$ to $1.94 \cdot 10^6$ items), compared to the total prescribing volume for the complete collection of drugs, which augmented of 17.5%. In the same period, the English population augmented of 7.6%[1]. Correcting for OME, it is possible to realize that effectively dispensed opioids were more marked, with an increment of 41% (from $1.61 \cdot 10^9$ to $2.27 \cdot 10^9$ OME milligrams), as we can observe in Figure 1. However, from late 2016, in both opioid prescriptions and total OME amount a slight drop in trend started. This is consistent with the introduction in 2016 of the Opioids Aware

[1]ONS: Vital statistics in the UK: births, deaths and marriages

Resource[2] that is a recommendation for patients and healthcare professionals to support prescribing of opioid medicines for pain.

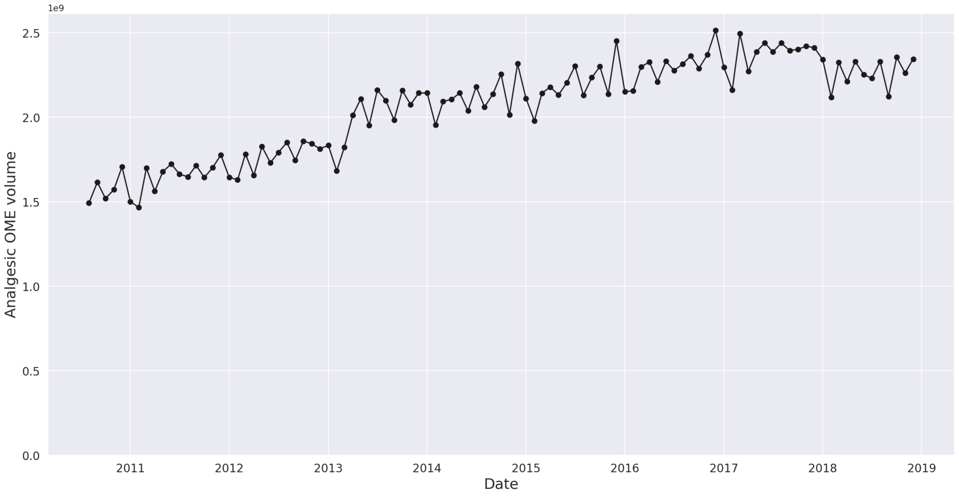

Figure 1: Prescribed OME volume in England.

[2] `https://fpm.ac.uk/faculty-of-pain-medicine/opioids-aware`
