# Supplementary figures and images for: Spatial heterogeneity and socioeconomic determinants of opioid prescribing in England between 2015 and 2018
Source: BMC Med. 2020 May 15;18:127. doi: 10.1186/s12916-020-01575-0 (PMC7227089; doi:10.1186/s12916-020-01575-0)

## Additional File 3

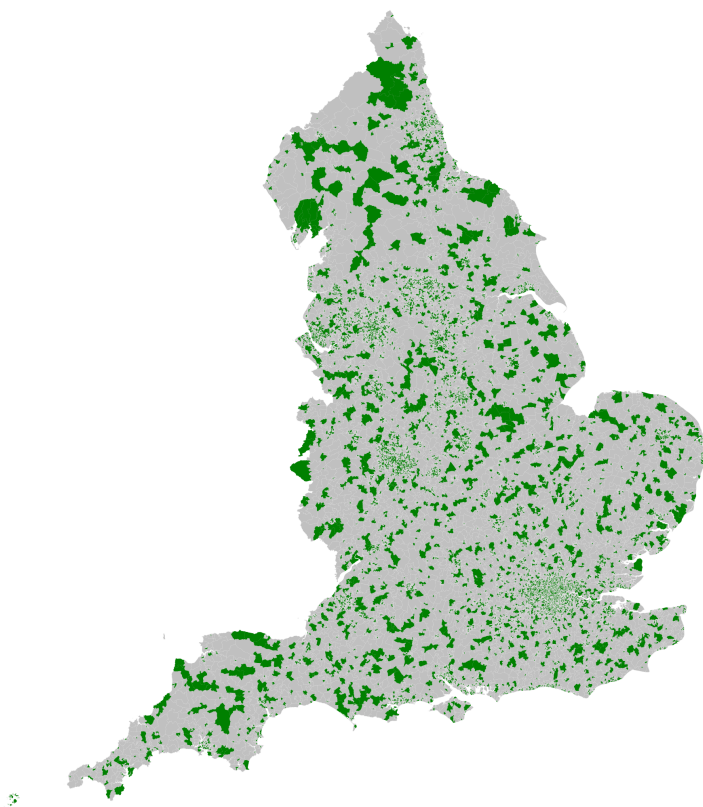

Figure 1: LSOAs with at least a general practice in England.

Supplement: Supplementary file 3 — Additional file 3 Spatial distribution of the general practices in England at LSOA level. [file 12916_2020_1575_MOESM3_ESM.pdf]
