# Supplementary material for: Spatial heterogeneity and socioeconomic determinants of opioid prescribing in England between 2015 and 2018
Source: BMC Med. 2020 May 15;18:127. doi: 10.1186/s12916-020-01575-0 (PMC7227089; doi:10.1186/s12916-020-01575-0)
Supplement: Supplementary file 4 — Additional file 4 Sensitivity analysis of the local measures of spatial association according to different weighting schemes. [file 12916_2020_1575_MOESM4_ESM.pdf]

Additional File 4

Sensitivity analysis of local measures of spatial association

We have extended the original experimental settings with a sensitivity analysis to address the effect of the choice of the weighting scheme. We implemented the following alternatives: rook, queen, k-nearest neighbors (with k=[5, 10, 15, 20]), kernel functions with adaptive bandwidth (uniform and gaussian forms) where we explore k=[5, 10, 15, 20] as the neighborhood size to compute the bandwidth. A Euclidean distance function between centroids of the spatial units has been used to rank areas by distance.

Table 1 presents a summary of the distribution of the resulting significative areas while Figure 1, Figure 2, Figure 3, and Figure 4 show their spatial configuration on choropleth maps. As expected, we observe that higher neighborhood size for distance-based methods results in a broader set of significant areas retrieved. It is worth noting that for the same weighting, the set of areas with a specific k is a subset of the areas retrieved with $k_2 > k$. Moreover, weighting schemes with comparable neighborhood size identify similar areas independently of the weighting function. In fact, the areas overlap between knn_k, $gaussian_k$, and $uniform_k$ with the $k = [5, 10, 15, 20]$ ranges between 0.846 and 0.998. The overlap between the reference scheme *queen* and the alternatives with $k = 5$ (this is an approximation that uses k ≈ average number of neighbors in a queen weighting) is, respectively, 0.87 and 0.94 for knn and gaussian/uniform.

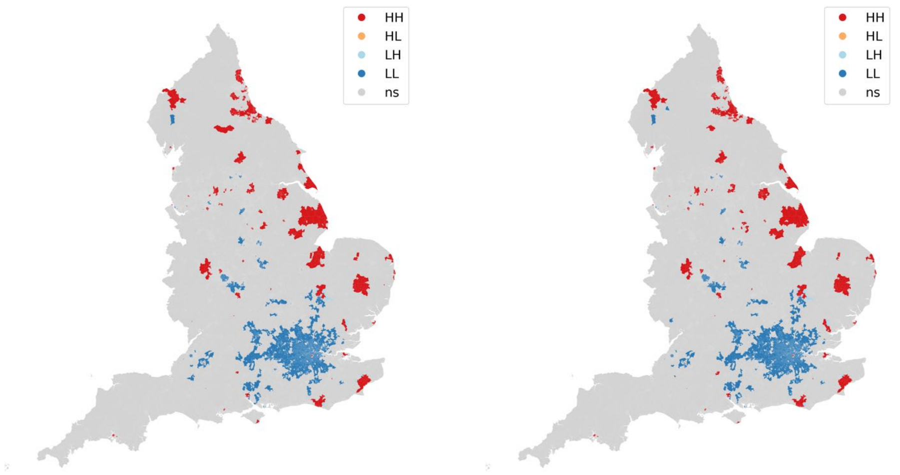

(a) rook (b) queen

Figure 1: `contiguity`

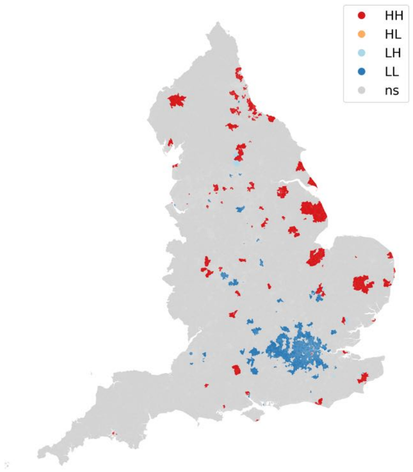

(a) k=5

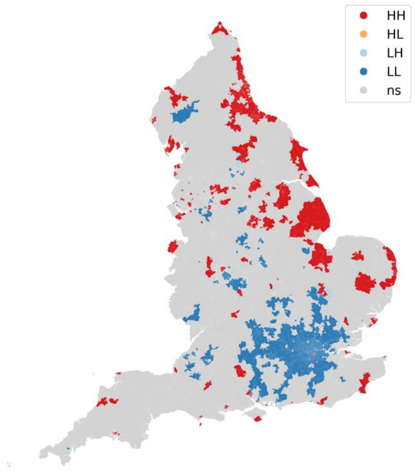

(b) k=10

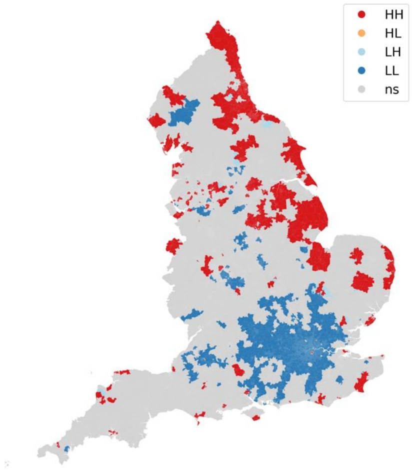

(c) k=15

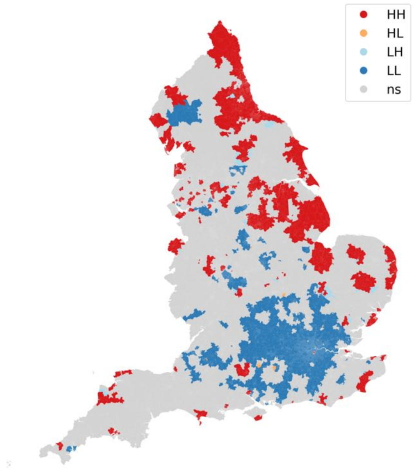

(d) k=20

Figure 2: `knn`

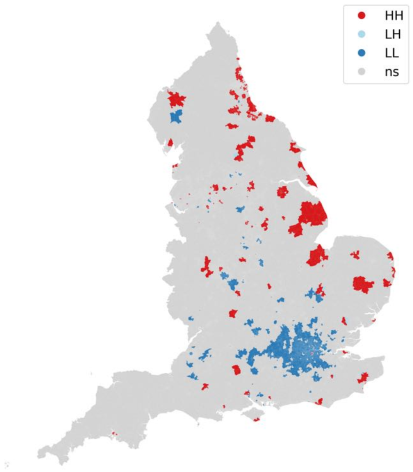

(a) k=5

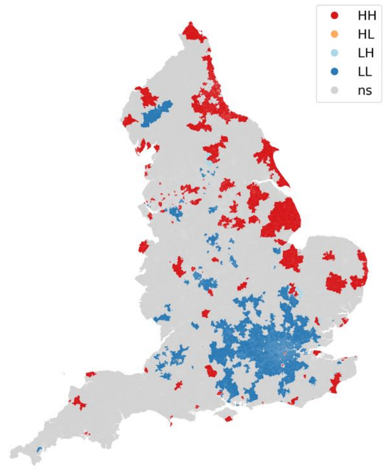

(b) k=10

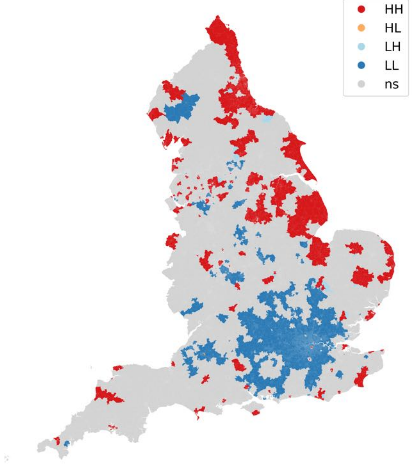

(c) k=15

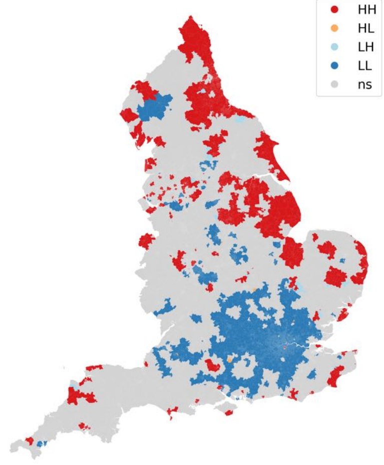

(d) k=20

Figure 3: `gaussian`

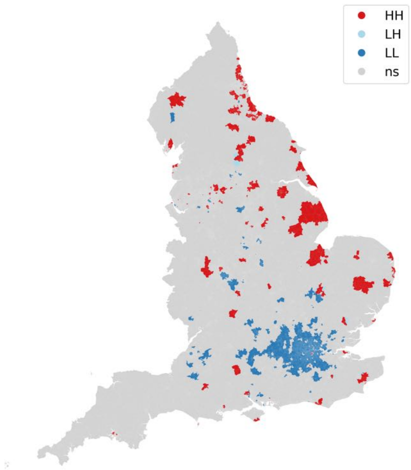

(a) k=5

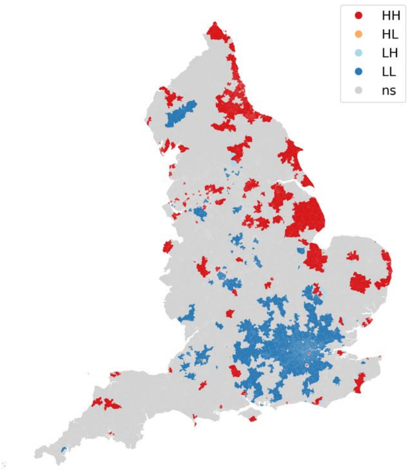

(b) k=10

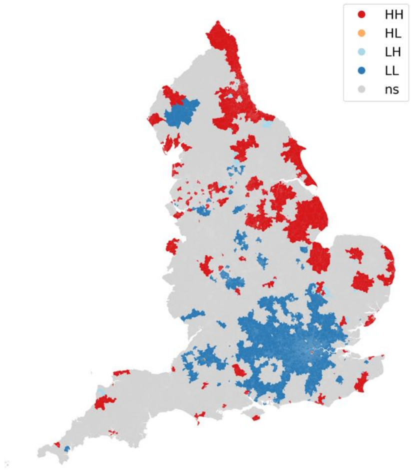

(c) k=15

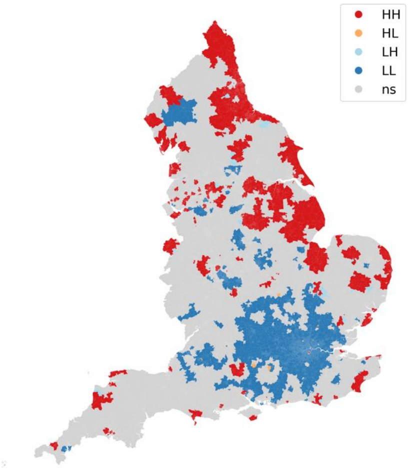

(d) k=20

Figure 4: `uniform`

Table 1: Statistics for LISA measures for different weighting schemes: the subscript represents the number of neighbors for the *knn* method and the size of the bandwidth for the adaptive kernel functions. The table contains the number of LSOA where the LISA with the FDR correction is significant, and the HH, LH, LL, and LL columns the number of high-high, low-high, low-low and low-low areas, respectively.

weight	LSOA	HH	LH	LL	HL
$rook$	7701	1145	31	6523	2
$queen$	7695	1138	29	6526	2
knn_5	7173	1049	22	6101	1
knn_{10}	11911	2866	52	8984	9
knn_{15}	13885	3705	76	10083	21
knn_{20}	15413	4402	124	10846	41
$uniform_5$	8478	1448	22	7008	0
$uniform_{10}$	12551	3135	49	9361	6
$uniform_{15}$	14337	3935	73	10314	15
$uniform_{20}$	15676	4516	116	11007	37
$gaussian_5$	8300	1374	20	6906	0
$gaussian_{10}$	12482	3121	45	9313	3
$gaussian_{15}$	14284	3905	67	10299	13
$gaussian_{20}$	15636	4484	102	11021	29
