# Supplementary material for: Spatial heterogeneity and socioeconomic determinants of opioid prescribing in England between 2015 and 2018
Source: BMC Med. 2020 May 15;18:127. doi: 10.1186/s12916-020-01575-0 (PMC7227089; doi:10.1186/s12916-020-01575-0)
Supplement: Supplementary file 5 — Additional file 5 Analysis of determinants at MSOA level (2018). [file 12916_2020_1575_MOESM5_ESM.pdf]

Additional File 5

Table 1: Median proportion of OME contained in MSOA grouped by quintiles (2018). Odds ratio and 95% CI provided by the multivariable logistic regression.

MSOA		
Quantile feature	High median	Multivariable Logit
ID employment		
least deprived	6.9%	1 (ref)
.	11.2%	3.16 (2.59-3.85)
.	14.1%	6.58 (5.32-8.14)
.	16.8%	21.75 (17.01-27.81)
most deprived	21.9%	93.2 (69.51-124.96)
% whites		
lowest percentage	3.9%	1 (ref)
.	9.8%	4.31 (3.21-5.79)
.	14.5%	8.52 (6.16-11.79)
.	18.7%	14.65 (10.4-20.65)
highest percentage	24.1%	33.62 (23.48-48.13)
ID housing		
least deprived	18.4%	1 (ref)
.	17.1%	0.85 (0.7-1.03)
.	17.7%	0.75 (0.61-0.92)
.	13.6%	0.44 (0.35-0.56)
most deprived	4.0%	0.22 (0.16-0.32)
% apprenticeship		
lowest percentage	3.2%	1 (ref)
.	10.2%	2.5 (1.84-3.38)
.	15.7%	3.71 (2.68-5.14)
.	19.5%	4.04 (2.89-5.64)
highest percentage	22.2%	4.61 (3.27-6.5)

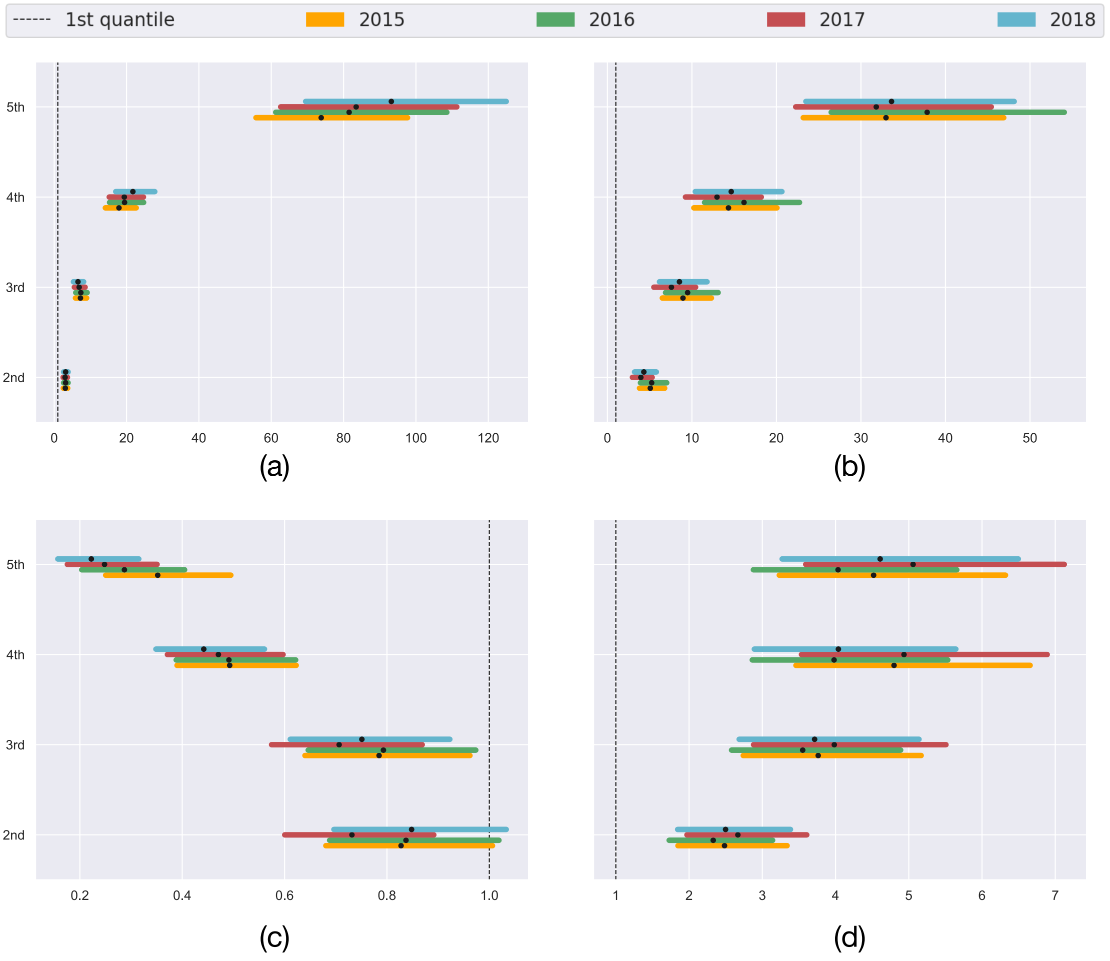

Figure 1: Odds ratio and 95% CI provided by the multivariable logistic regression at MSOA level for the predictors: (a) employment, (b) white, (c) housing, and (d) apprenticeship.
